# Proton-Conducting Sulfonated Periodic Mesoporous Organosilica

**DOI:** 10.3390/nano16030203

**Published:** 2026-02-04

**Authors:** Tobias Wagner, Michael Tiemann

**Affiliations:** Department of Chemistry, Paderborn University, 33098 Paderborn, Germany; tobias.wagner@uni-paderborn.de

**Keywords:** mesoporous organosilica, sulfonic acid functionalization, fuel cell membrane, proton conduction, humidity dependence, impedance, flexible spacer, mesopore ordering

## Abstract

Proton exchange membranes (PEMs) are essential for fuel cells, yet conventional materials like Nafion suffer from humidity dependence and limited thermal stability. This study introduces sulfonated phenylene-bridged periodic mesoporous organosilicas (PMOs) as promising inorganic–organic hybrid PEMs, synthesized via surfactant-templating with varying alkyl chain lengths for different mesopore sizes. Post-synthetic functionalization involves nitration of phenylene moieties, reduction to amines, and ring-opening of propane or butane sultones to graft sulfonic acid groups via flexible spacers, achieving homogeneous distribution along pore walls. Post-functionalization is confirmed by powder X-ray diffraction (PXRD), revealing preserved 2D hexagonal p6mm ordering and phenylene stacking. N_2_ physisorption shows type IV isotherms with reduced pore volumes and pore sizes. ^1^H NMR is used to quantify functionalization degrees. Impedance spectroscopy on pressed pellets demonstrates proton conductivities up to 2 × 10^−3^ S cm^−1^ at 30 °C and 90% RH, depending on the functionalization degree, confirming sulfonic acid-mediated conduction.

## 1. Introduction

Fuel cells represent a compelling pathway towards a sustainable energy future, offering high efficiency and low emissions [1]. A critical component of most fuel cell designs is the proton exchange membrane (PEM), responsible for selective proton transport and electrical insulation between the anode and cathode [2]. Nafion, a perfluorosulfonic acid polymer, remains the dominant PEM material so far. It consists of a hydrophobic fluorinated main chain with hydrophilic sulfonic acid side chains. When hydrated, Nafion exhibits high proton conductivity due to pathways created by phase separation between the hydrophobic backbones and hydrophilic clusters [3]. However, its dependence on humidification for optimal performance and its limited high-temperature stability pose significant challenges for broader implementation. To overcome these drawbacks, research has expanded beyond conventional polymeric membranes towards inorganic–organic hybrid materials.

Mesoporous silica-based materials, with their high surface areas and tunable pore structures, present an attractive architecture for a multitude of applications in various fields like catalysis [4,5,6], energy conversion [7], and medicine [8,9]. Among them, organic–inorganic hybrid silica-based materials are particularly promising [10]. In particular, periodic mesoporous organosilicas (PMOs) [11,12,13,14] stand out for their excellent functionalization possibilities. Introducing functional groups into porous silica is a widely employed strategy to create solid acid catalysts 5] and proton conductors [15,16,17,18,19,20,21]. Sulfonic acid groups (–SO_3_H) are most widely used to increase proton conductivity inside the pore channels because of their high acidity and hydrophilicity, characteristics needed for proton mobility. The pore structure, density, and accessibility of their functional groups are crucial determinants of proton conductivity [22,23,24].

In this study, we investigate phenylene-bridged PMOs functionalized with sulfonic acid groups attached to the PMO backbone through carbon chains (spacers) of different lengths. The concept relies on the homogeneous distribution and well-defined orientation of the organic (phenylene) moieties along the pore walls, suggesting the possibility of an equally homogeneous distribution of the sulfonic acid functions. At the same time, the PMO material is less hydrophilic than pure mesoporous silica materials [25], which is advantageous for proton conductivity mediated by the sulfonic acid groups, similar to Nafion. We use a post-synthetic method to create homogeneously distributed functional groups at the pore walls, varying pore size, spacer length, and the degree of functionalization, allowing us to study their influence on proton conductivity. Optimization of these parameters led to proton conductivity up to 2 × 10^−3^ S/cm (at 30 °C and 90% r.h.), almost reaching that of Nafion, demonstrating comparability to other optimized silica-based materials. In addition, the materials hold promise for application as solid acid catalysts or for the ion exchange-based separation and purification of charged molecules.

## 2. Materials and Methods

### 2.1. Synthesis

The PMOs were synthesized similarly to the procedure first reported by Inagaki et al. [10]. In a typical synthesis procedure, 4.4 g of hexadecyltrimethylammonium bromide (CTABr) surfactant and 2.28 g of NaOH are dissolved at room temperature in 150 mL deionized water. Then, 6 mL of 1,4-bis(triethoxysilyl)benzol (BTEB) is added under vigorous stirring. The mixture is stirred for 24 h at room temperature and then placed in an oven at 80 °C for another 24 h. The solid product is filtered off and dried bevor extraction of the template with a mixture of ethanol and HCl using Soxhlet apparatus. In our study, to vary pore size, corresponding amounts of other alkyltrimethylammonium bromide surfactants (dodecyl, tetradecyl, octadecyl) were used instead of CTABr [26]. Samples were named PMO-12, -14, -16, and -18 according to the length of the hydrocarbon chain in the surfactant.

### 2.2. Functionalization

The PMOs were functionalized through a procedure based on sultones [27]. To this end, the phenylene ring was nitrated, followed by the reduction of the nitro group to the amino function, which then reacted with 1,3-propane sultone or 1,4-butane sultone. In the procedure, 1 g of PMO was added to a mixture of 25 mL sulfuric acid (95%) and 10 mL nitric acid (65%) and stirred for 72 h at room temperature. Then, it was poured into 300 mL of ice water. The solid product was filtered off, washed with water, and dried. The powder was dispersed in 30 mL of HCl (37%), and 3 g of SnCl_2_ was added as a reducing agent. The mixture was stirred at room temperature for 72 h. Then, it was diluted with ice water, filtered, washed, and dried. To attach the sulfonic acid groups, 0.5 g of the amino-functionalized PMO was dispersed in 50 mL cyclohexane and 0.5 mL of sultone was added. The reaction was stirred for 72 h at 40 °C. Samples were named -R1 for the shorter (C_3_) and -R2 for the longer (C_4_) spacer group between the phenylene backbone and the amino group (resulting from the 1,3-propane and the 1,4-butane sultone, respectively).

### 2.3. Characterization

Powder X-ray diffraction (PXRD) was performed on a Bruker D8 Advance diffractometer (Bruker, Karlsruhe, Germany) (0.55° ≤ 2θ ≤ 50°) with a step size of 0.02° and a counting time of 3 s per step. A Cu-Kα X-ray source (λ = 0.154 nm) was used. For N_2_ and H_2_O sorption studies, samples were degassed at 120 °C for 12 h in vacuum prior to measurements. N_2_ physisorption was performed with a Quantachrome Instruments Autosorb 6B instrument (Quantachrome Instruments, Boca Raton, FL, USA) at 77 K. Pore size was estimated using the BJH method on the desorption branch, with total pore volume calculated as p/p_0_ = 0.99. H_2_O sorption experiments were performed with a Micromeritics 3Flex instrument (Micromeritics, Unterschleißheim, Germany) at 293 K. IR measurements were performed in transmission mode (KBr pellets) with a Bruker Vertex 70 FT-IR spectrometer (Bruker, Ettlingen, Germany). NMR spectra were measured on a Bruker Ascent 700 instrument (Bruker, Ettlingen, Germany). Samples were dissolved in 40 w% NaOD in D_2_O over several days. The functionalization degrees were calculated by integrating the signals of the protons in different chemical environments. Thermogravimetric analysis (TGA) with ion mass detection (MS) was performed with a Linseis PT1000 thermobalance (Linseis, Selb, Germany) in synthetic air, with a heating rate of 10 K/min. A Pfeiffer Vacuum Omnistar mass spectrometer (Pfeiffer Vacuum, Aßlar, Germany) was connected to the balance. Proton conductivity was assessed by performing impedance measurements. Prior to the measurements, the samples were pressed into cylindrical pallets with a 13 mm diameter and 1 mm thickness without additives, using a pressure of 2 t. Samples were placed between two gold-plated electrodes. Impedance data were recorded using a Novocontrol broadband dielectric spectrometer (Alpha-A High Performance Frequency Analyzer) (Novocontrol Technologies, Montabaur, Germany). The frequency range was 1 Hz to 4.6 MHz at an applied voltage of 0.1 V. Proton conductivity *σ* was calculated from the resistance *R* by accounting for the thickness *L* and contact area *A* of the sample pellet between the two electrodes (*σ* = *R*^−1^ × *L* × *A*^−1^). For temperature and humidity control, measurements were carried out inside an Espec SH-242 climate chamber. Prior to each measurement, samples were equilibrated for 24 h.

## 3. Results and Discussion

### 3.1. Non-Functionalized PMOs

The powder X-ray diffraction (PXRD) patterns of the non-functionalized materials (after surfactant extraction) show the expected features of phenylene-bridged PMOs (Supplementary Materials, Figure S1). Reflections in the low-angle region confirm the 2D hexagonal (p6mm) pore arrangement. The corresponding lattice parameter depends on the respective chain length of the surfactant used as the porogenic structure director in the synthesis [25]; their values are 3.4 nm (C_12_), 3.9 nm (C_14_), 4.2 nm (C_16_), and 4.5 nm (C_18_). In the following, samples are labeled as PMO-*n*, where *n* = 12, 14, 16, 18 denotes the chain length of the surfactant. Additionally, the materials show three PXRD reflections in the wide-angle region, confirming the periodically ordered arrangement of the phenylene groups inside the pore walls, with a spacing of 0.76 nm; this is independent of the surfactant used [28].

N_2_ adsorption–desorption isotherms are also shown in the Supplementary Materials (Figure S2). The choice of surfactant has a significant impact. While all materials exhibit isotherms of mostly type IV nature, the C-12 material shows similarities to a type I isotherm, attributable to very small mesopores. On the other hand, the C-18 PMO shows the rise of the isotherm at higher partial pressures, a trend commonly seen for type II isotherms, indicating the presence of large inter-particle voids. The pore size distribution curves (Figure S2, insert) show a clear correlation between surfactant chain length and pore size [27].

### 3.2. Characterization of Functionalized Samples

**Figure 1:**
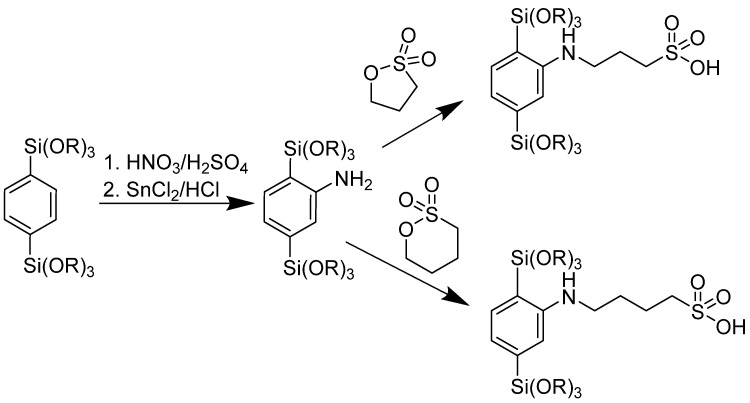
A schematic of the functionalization of the PMOs.

Functionalization of the PMO materials was carried out in a three-step process as shown in Figure 1. The phenylene rings in the pore walls were nitrated, followed by the reduction of the nitro group to the amino group. In the last step, 1,3-propane sultone or 1,4-butane sultone (i.e., cyclic sulfonic acid esters) reacted with the amino group, attaching the sulfonic acid function though flexible linkers (spacers) of three or four methylene groups.

**Figure 2:**
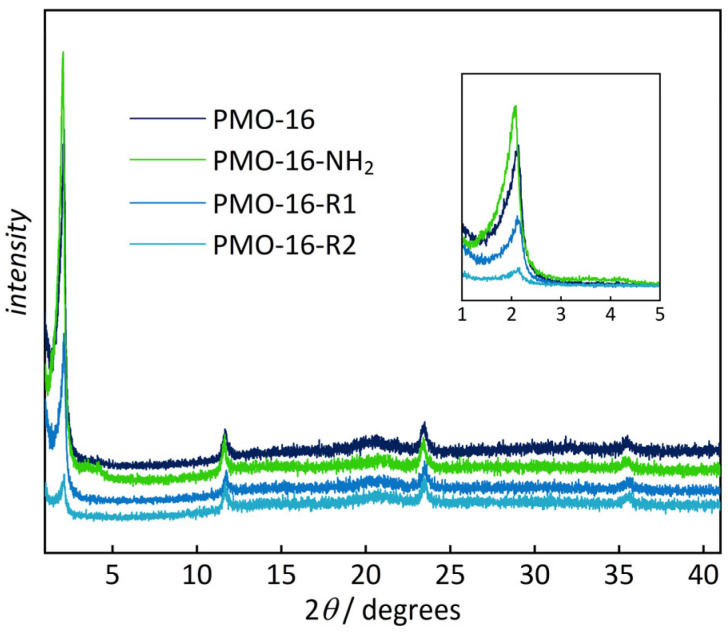
Powder XRD patterns for the functionalization of PMO-16. Data for samples with other pore sizes are shown in Figure S3.

**Figure 3:**
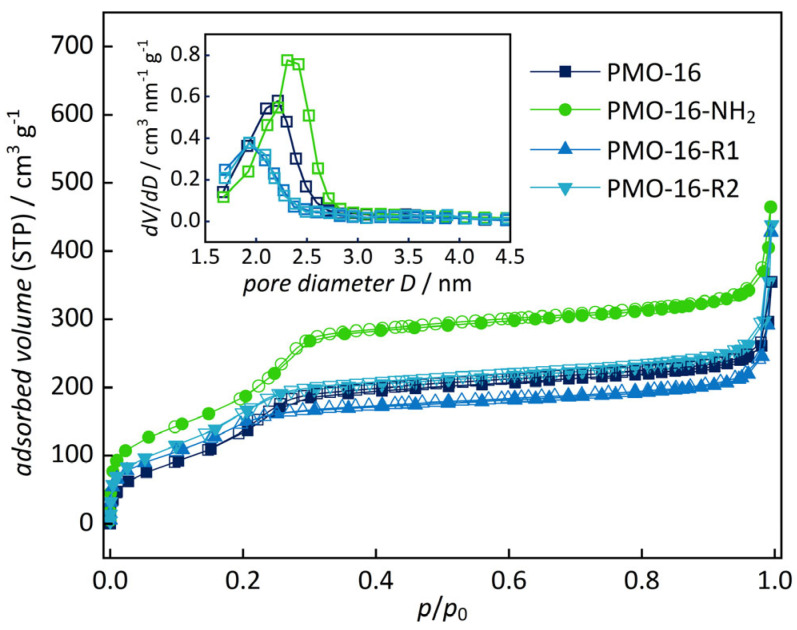
N_2_ physisorption isotherms and BJH pore size distributions (inset) of functionalized PMO-16. Closed symbols represent the adsorption, open symbols the desorption branch. (Data for samples with other pore sizes are shown in Figure S4).

PXRD diagrams (Figure 2) confirm the structural integrity of the functionalized samples (see also Supplementary Materials, Figure S3). The periodic pore arrangement is slightly less ordered after the addition of the sulfonic acid groups, as indicated by the decrease in intensity of the low-angle reflection. In contrast, the periodic stacking of the phenylene rings in the pore walls remains, as apparent from reflections in the wide-angle region. N_2_ physisorption analysis (Figure 3; see also Supplementary Materials, Figure S4 and Table S1) confirms the porosity of the functionalized samples. The functionalized samples exhibit type IV isotherms, resembling their unfunctionalized predecessors. As expected, the specific pore volumes and the pore sizes are reduced after functionalization, caused by partial filling of the pores with the spacer and sulfonate group. No hysteresis is observed, indicating homogeneous functionalization. Pore volumes and pore sizes are slightly larger for the amino-functionalized samples than for the final sulfonic acid-functionalized materials. This may be explained by residuals of the surfactant being removed during the acid treatment in the final step.

Samples were characterized by FTIR spectroscopy (see Supplementary Materials, Figure S5). All spectra are dominated by the strong Si–O vibrations at 1070 cm^−1^. The aromatic C–C bonds are also visible for all samples at 1630 cm^−1^. For the nitrated samples, two strong bands appear, corresponding to the N–O vibrations at 1350 cm^−1^ and 1540 cm^−1^ as well as a weaker C–N vibration band at 1280 cm^−1^. As expected, the N–O bands disappear with the reduction of the nitro groups. For the sulfonic acid-functionalized samples, a new shoulder at 1200 cm^−1^ confirms the presence of a sulfonic acid group [29,30].

The thermal stability of the materials was investigated by thermogravimetric analysis (TGA) with ion mass detection (MS) in air (see Supplementary Materials, Figures S6 and S7). Mass fragments with *m*/*z* = 18 (H_2_O^+^), *m*/*z* = 44 (CO_2_^+^), and *m*/*z* = 64 (SO_2_^+^) were tracked. All samples exhibit three distinguishable decomposition steps. The first one up to 100 °C is attributed to the loss of residual intra-pore water. The second step starting at ca. 200 °C is related to the decomposition of the sulfonate (detection of SO_2_^+^). Starting at ca. 500 °C, the final and largest mass loss step corresponds to the decomposition of the organic moieties (detection of CO_2_^+^). We conclude that the materials are stable up to 200 °C, allowing for high-temperature application in fuel cells [31].

For quantitative assessment of the degree of functionalization, ^1^H NMR spectra of samples dissolved in NaOD/D_2_O were recorded (see Supplementary Materials, Figure S8, for the spectra of the PMO-16 materials as examples). For the non-functionalized samples, a singlet is observed at 6.5 ppm, which corresponds to the four identical H atoms of the phenylene ring. Additional peaks in the form of a triplet at 0 ppm and a quartet at 2.4 ppm can be attributed to incomplete hydrolysis of the ethoxy groups. For the amino-functionalized samples, a set of three additional peaks occurs, one singlet and two doublets with identical integrals. This pattern is consistent with single functionalization of the phenylene ring. Peaks corresponding to ethoxy groups have disappeared, indicating complete hydrolysis. The degree of functionalization was calculated based on the fraction of the protons corresponding to the amino-functionalized and the unfunctionalized rings. Therefore, 100% functionalization is defined as full conversion of the phenylene rings with one amino function each (see Supplementary Materials, Equation (S1)). No further impurities are observed, indicating complete reductive conversion of the nitro groups and no double nitration.

After reaction with the sultones, peaks corresponding to aliphatic H atoms occur. These can be divided into two groups, one containing the inner and the other containing the outer methylene groups in the spacer. Accordingly, the integrals show a ratio of 2:1 for the propylene and 1:1 for the butylene spacer, respectively [32]. The ratio of aliphatic to aromatic protons is used to calculate the overall degree of functionalization, as described in the Supplementary Materials (Equations (S2)–(S4)). This was used to calculate the ion exchange capacity as well as the functionalization density. The results are shown in Table 1.

There is a significant difference in the degree of functionalization among the samples. Regarding amino group functionalization, sample PMO-14 stands out with the highest degree of 60%. The other samples show little variance, ranging from 35% to 43%, with no apparent trend related to pore size. For the sulfonate group-functionalized materials, in general, the larger 1,4-butane sultone leads to a significantly lower functionalization degree than the smaller 1,3-propane sultone [26]. This may be attributable to the larger sultone size limiting diffusion in the mesopore channels and causing more steric hindrance, as well as to its lower reactivity. The C18-PMO shows lower functionalization with both sultones even though the degree of amino functionalization is medium. In contrast, the C16-PMO shows the highest degree of sulfonate functionalization but the lowest degree of amino functionalization. Sulfonate functionalization of more than 100% may indicate that some amino groups have reacted with two sultones (resulting in tertiary instead of secondary amino functions). Other possible reasons might be unreacted sultones or solvent molecules residing in the pores. Interestingly, this is only seen for the sample with the lowest density of amine groups, underlining the large space needed for the aliphatic spacer and the sulfonate head. Overall, the density of sulfonate groups is similar to other known functionalized (organo)-silica compounds and Nafion [20,22,33,34].

**Figure 4:**
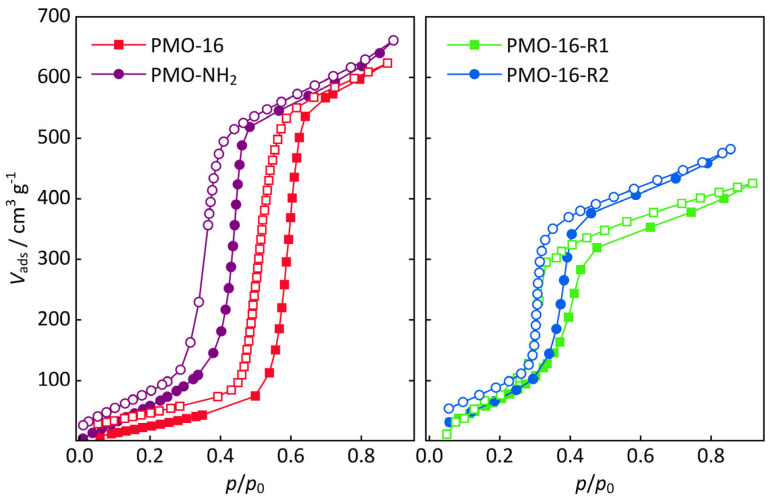
Water vapor sorption isotherms for the C16-PMO series (**left**: PMO-16—non-functionalized, PMO-NH_2_—amino-functionalized; **right**: PMO-16-R1 and -R2—sulfonic acid group-functionalized with different spacer lengths). Closed symbols represent the adsorption, open symbols the desorption branch.

Water vapor sorption isotherms are shown in Figure 4. All samples show isotherm shapes typical of water sorption on mesoporous adsorbents. After amino group functionalization, the point of pore condensation shifts to lower relative pressure and the slope of the isotherm before condensation is increased (left figure). This can be attributed to an increase in hydrophilicity [35,36]. The effect is even more pronounced for the sulfonate group-functionalized samples (right figure), facilitating water condensation, which is relevant in proton conduction (see below).

### 3.3. Proton Conductivity

To investigate the proton conductivity of the sulfonate group-functionalized PMO materials, we pressed pellets without the use of additives. The applied pressure was kept as low as possible to achieve stable pallets while keeping the structure intact. After pressing, physisorption analysis revealed a slightly reduced surface area of about 20%. Impedance was measured in the frequency range of 1 Hz to 4.6 MHz. An equivalent circuit consisting of a resistor and a parallel combination of a constant-phase element (CPE) and another resistor (Figure S9) was fitted to the semicircles (using ZView 4 software) [37]. The resistor parallel to the CPE is considered to correspond to the intrinsic proton resistance (i.e., reciprocal conductivity) of the material; the single resistor accounts for the contact resistance between the electrodes and the sample. Nyquist diagrams are shown in the Supplementary Materials (Figure S10). All samples exhibit depressed semicircles in the high-frequency region that are in good agreement with the fit. In some of the samples, an additional semicircle of varying separation is visible. This may be caused by inhomogeneous functionalization of the pores and/or conduction outside of the pores between particles [38]. The second semicircle is generally more pronounced in the PMO-R2 samples with the longer spacers. For data evaluation, only the high-frequency semicircle was fitted. The observed proton conductivity is expected and can be explained by the presence of the strongly acidic sulfonic acid function, similar to Nafion. In addition, the strong hydrophilicity observed by water vapor sorption (see above) will further mediate proton conduction.

**Figure 5:**
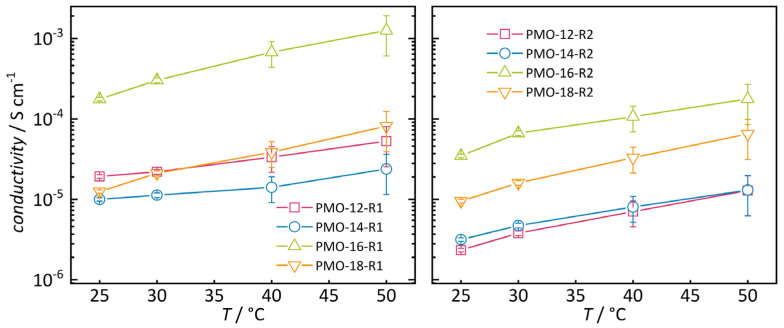
Conductivities of samples R1 (**left**) and R2 (**right**) calculated from fitting impedance data.

All sulfonate group-functionalized samples show a clear increase in proton conductivity with increasing temperature (Figure 5). In general, the materials with a shorter spacer between the phenylene backbone and sulfonate group exhibit higher conductivity. No clear trend of conductivity versus PMO pore size is observed. Only the PMO-16 sample stands out by having the highest conductivity with both spacer lengths [25]. This is explainable by the fact that the materials from the PMO-16 series possess a higher degree of mesoscopic structural order than the other samples, as apparent from narrower low-angle powder XRD peaks (Figure S3) and sharper pore size distribution (Figure S4).

To analyze the impact of functionalization degree, a series of samples based on PMO-16 were prepared using varying relative amounts of propane sultone. As before, functionalization degrees were calculated from NMR data, ranging from 40% to 137% (sulfonate functions) (Table 2). Again, functionalization degrees of more than 100% indicate that some amino groups have reacted with two sultones.

**Figure 6:**
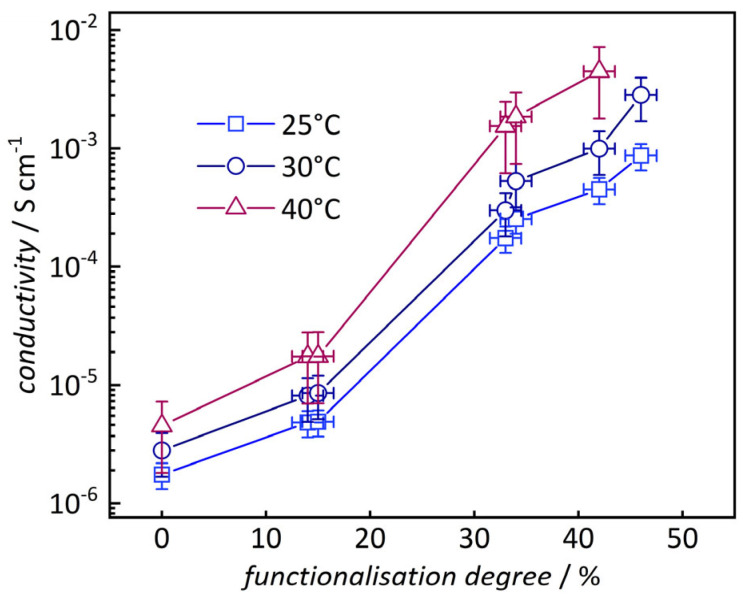
Conductivity of samples with different functionalization degrees measured at differing temperatures and 90% r.h.

The results show a strong increase in conductivity with increasing functionalization degree from 0% to 46% by more than two orders of magnitude up to 2 × 10^−3^ S cm^−1^ (at 90% r.h., Figure 6 and Figure S11). While the values are lower than those reported for Nafion, the conductivity is comparable to other optimized silica-based materials (see Table S2) [21,23,24,39,40,41]. This effect is apparent at all temperatures employed. This clearly confirms that proton conduction occurs predominantly through the sulfonate groups [22].

**Figure 7:**
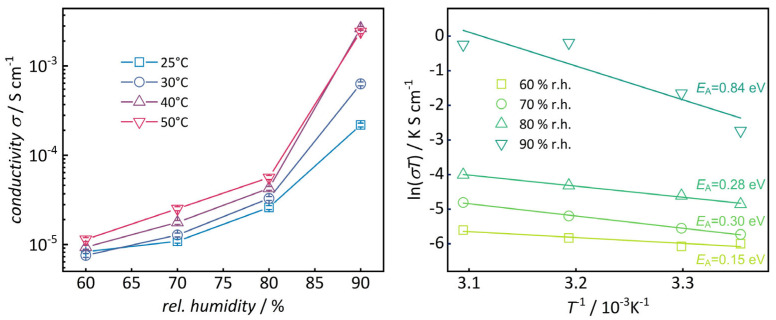
(**Left**) Conductivity at varying temperature and humidity. (**Right**) Arrhenius plots.

To assess the impact of humidity, the conductivities of the sample with the highest degree of functionalization (46%) were measured at relative humidities ranging from 60% to 90%. As is known in the literature, humidity has a strong impact on sulfonate-mediated proton conductivity [42]. From 60% r.h. to 90% r.h., an increase of more than two orders of magnitude is observed (Figure 7(left) and Figure S12). Higher humidity was not evaluated to avoid water condensation in the climate chamber [22]. The activation energy *E*_A_ was calculated from Arrhenius plots (Figure 7(right)). Up to 80% r.h., the low values (<0.3 eV) indicate a conductivity mechanism that is predominantly governed by ‘proton hopping’ similar to the Grotthuß mechanism [43]. The significantly higher activation energy at 90 °C (0.84 eV) suggests a higher contribution of mass transport-related proton conduction.

## 4. Conclusions

Sulfonated phenylene-bridged periodic mesoporous organosilicas (PMOs) were synthesized with varying pore sizes using surfactants of different alkyl chain lengths. Post-synthetic functionalization proceeded via nitration of the phenylene rings, reduction to amino groups, and reaction with 1,3-propane or 1,4-butane sultones, yielding homogeneous sulfonic acid distribution along the pore walls. Powder X-ray diffraction confirms preservation of the 2D hexagonal p6mm pore ordering and 0.76 nm phenylene stacking after functionalization. N_2_ physisorption reveals type IV isotherms with reduced pore sizes/volumes and no hysteresis, indicating uniform modification. ^1^H NMR analysis of the digested samples quantifies amino functionalization degrees of 35–61% and sulfonate functionalization degrees up to 137% (PMO-16-R1), with sulfonate densities of 0.88–2.71 nm^−2^, exceeding those of Nafion. Water vapor sorption isotherms show increased hydrophilicity after sulfonation, with pore condensation at lower pressures. Impedance spectroscopy on pressed pellets demonstrates proton conductivities increasing with temperature, reaching 2 × 10^−3^ S cm^−1^ at 30 °C and 90% r.h. Conductivity rises over two orders of magnitude with sulfonate functionalization degree. Arrhenius plots reveal activation energies of 0.3 eV up to 80% r.h., consistent with proton hopping, and 0.84 eV at 90% r.h. These results highlight the influence of pore size, spacer length, and functionalization degree on proton conductivity in PMOs. Future work will have to be dedicated to comprehensive investigation of single-cell performance and application design, including the measurement of polarization curves, peak power densities, and open-circuit voltage curves [44].

## Figures and Tables

**Table 1:** Degree of functionalization as calculated from NMR data.

Sample	Amino Function [%]	Sulfonate Function [%]	Overall [%]	Ion Exchange Capacity [mmol/g]	Functionalization Density [nm^−2^]
PMO-12-R1	42 ± 1.5	90 ± 2.6	38 ± 1.4	1.64	3.77
PMO-14-R1	60 ± 1.5	77 ± 2.6	46 ± 1.4	1.89	4.84
PMO-16-R1	35 ± 1.5	111 ± 2.6	39 ± 1.4	1.68	1.94
PMO-18-R1	40 ± 1.5	55 ± 2.6	22 ± 1.4	1.04	1.65
PMO-12-R2	43 ± 1.5	58 ± 2.6	25 ± 1.4	1.13	2.06
PMO-14-R2	61 ± 1.5	46 ± 2.6	28 ± 1.4	1.24	2.71
PMO-16-R2	36 ± 1.5	65 ± 2.6	24 ± 1.4	1.09	1.10
PMO-18-R2	41 ± 1.5	33 ± 2.6	13 ± 1.4	0.65	0.88

**Table 2:** Functionalization degrees and densities.

Sample	Amino Function [%]	Sulfonate Function [%]	Overall [%]	A_BET_ [m^2^/g]	Ion Exchange Capacity [mmol/g]	Functionalization Density [nm^−2^]
1	36 ± 1.5	40 ± 2.6	14 ± 1.4	660	0.70	0.64
2	32 ± 1.5	47 ± 2.6	15 ± 1.4	709	0.70	0.60
3	35 ± 1.5	95 ± 2.6	33 ± 1.4	529	1.44	1.63
4	35 ± 1.5	111 ± 2.6	39 ± 1.4	521	1.68	1.94
5	33 ± 1.5	125 ± 2.6	42 ± 1.4	522	1.73	2.00
6	34 ± 1.5	137 ± 2.6	46 ± 1.4	512	1.87	2.16

## Data Availability

Data are available from the authors upon request.

## Supplementary Materials

The following supporting information can be downloaded at https://www.mdpi.com/article/10.3390/nano16030203/s1: Figure S1: Powder XRD diagrams of the synthesized PMO materials after removal of the surfactants. Figure S2: N_2_ sorption isotherms of the synthesized PMO materials. Figure S3: Powder XRD patterns for functionalized samples with different pore sizes. Figure S4: N_2_ sorption isotherms of functionalized samples. Figure S5: FTIR spectra of the functionalized PMO-16 series. Figure S6: TGA-MS plots of functionalized PMO (R1). Figure S7: TGA-MS plots of functionalized PMO (R2). Figure S8: NMR spectra of digested samples. Figure S9: Equivalent circuit used for impedance data fitting. Figure S10: Nyquist plots for different pore sizes (left to right: PMO-12 to PMO-18) and different functionalization degrees (top: -R1; bottom: -R2); Figure S11: Nyquist plots for different functionalization degrees from 14% (top left) to 46% (bottom right); Figure S12: Nyquist plots for different humidities (top left—60 % r.h.; top right—70 % r.h.; bottom left—80 % r.h.; bottom right—90 % r.h.) at varying temperature; Table S1: N_2_ physisorption data of the samples; Table S2: Proton conductivity and IEC of comparable silica-based materials and Nafion [21,23,24,39,40,41].
