# Supplementary material for: Proton-Conducting Sulfonated Periodic Mesoporous Organosilica
Source: Nanomaterials (Basel). 2026 Feb 4;16(3):203. doi: 10.3390/nano16030203 (PMC12899766; doi:10.3390/nano16030203)
Supplement: Supplementary file 1 [file nanomaterials-16-00203-s001.zip › nanomaterials-4054774-supplementary.pdf]

Supplementary Materials

# Proton-Conducting Sulfonated Periodic Mesoporous Organosilica

**Tobias Wagner and Michael Tiemann ***

Department of Chemistry, Paderborn University, 33098 Paderborn, Germany; tobias.wagner@uni-paderborn.de
* Correspondence: michael.tiemann@upb.de

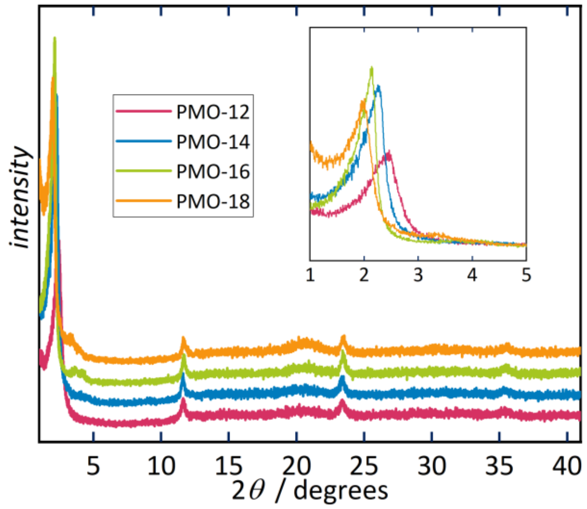


**Figure S1.** Powder XRD diagrams of the synthesized PMO materials after removal of the surfactants.

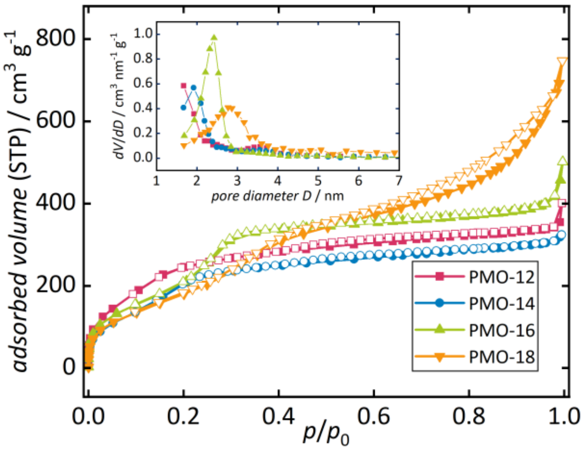


**Figure S2.** $N_2$ sorption isotherms of the synthesized PMO materials (filled symbols—adsorption, hollow symbols—desorption); BJH pore size distribution (inset).

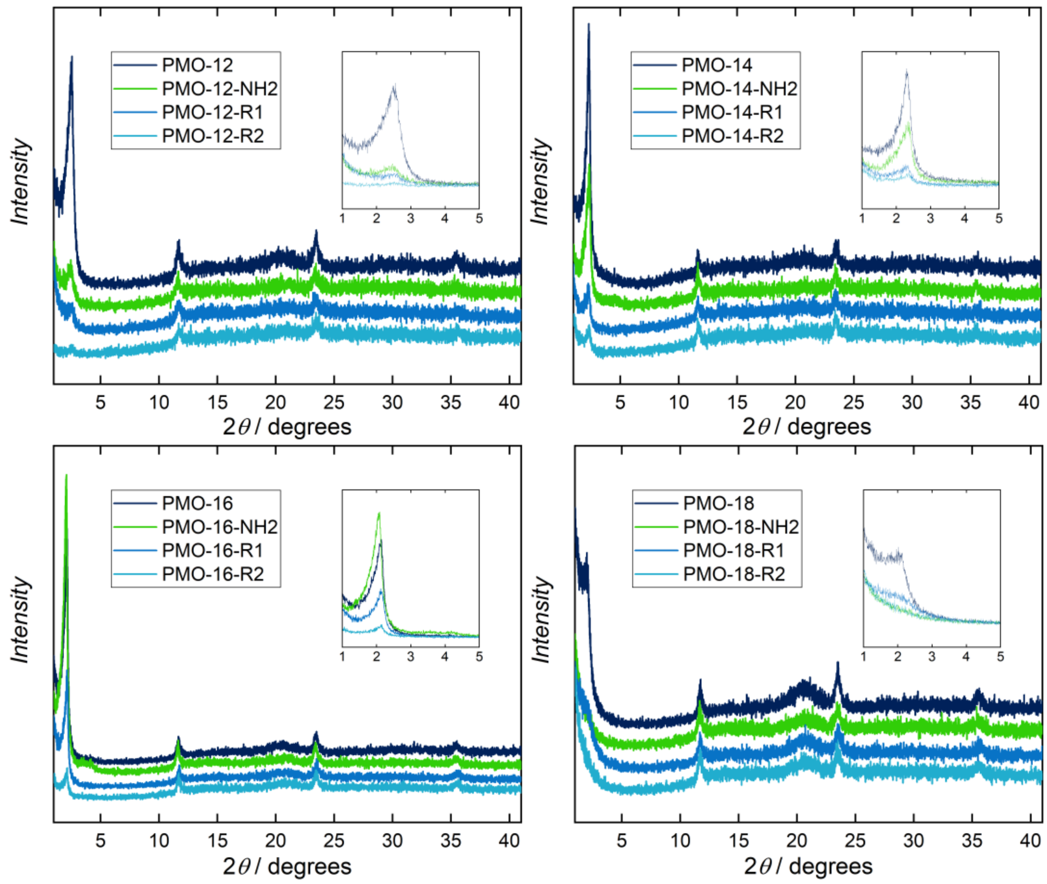


**Figure S3.** Powder XRD patterns for functionalized samples with different pore sizes.

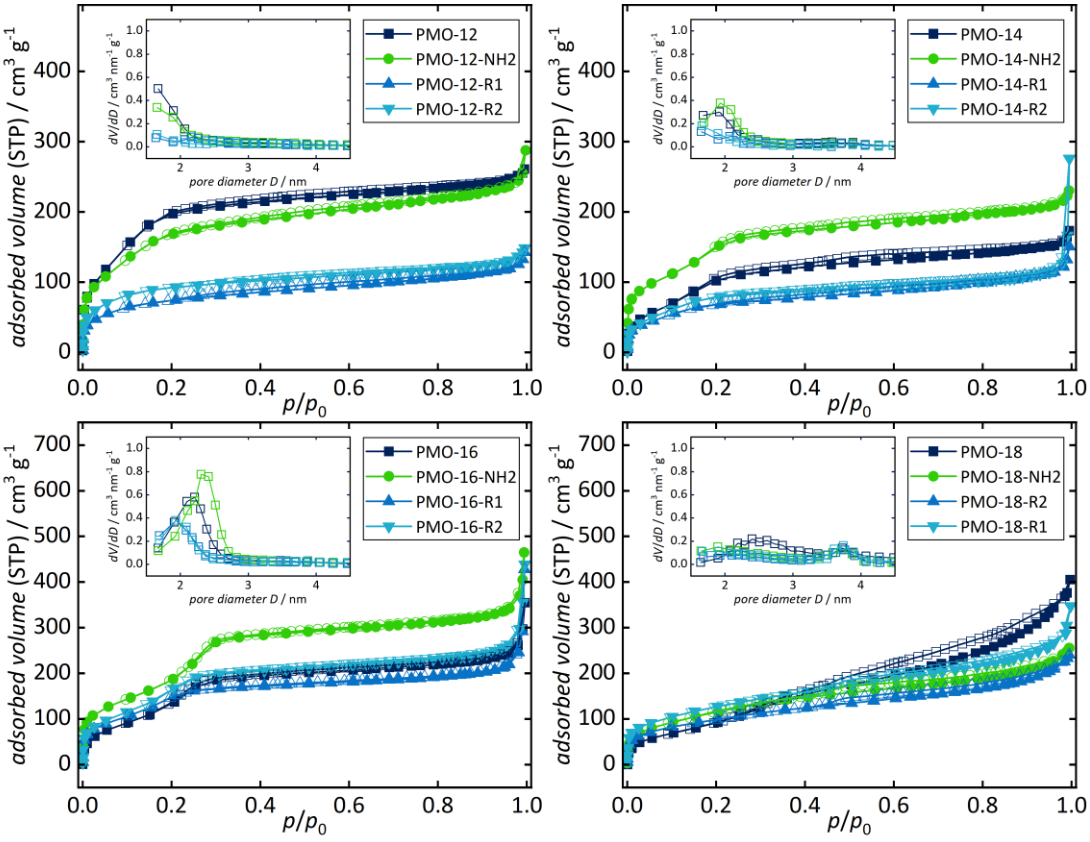


**Figure S4.** $N_2$ sorption isotherms of functionalized samples; BJH pore size distribution (inset).

**Table S1.** $N_2$ physisorption data of the samples.

| sample | pore size [nm] | $A_{BET}$ [m²/g] | specific pore volume [mL/g] |
|---|---|---|---|
| PMO-12 | <1.7 | 694 | 0.39 |
| PMO-14 | 1.92 | 395 | 0.25 |
| PMO-16 | 2.21 | 546 | 0.46 |
| PMO-18 | 2.40 | 381 | 0.58 |
| PMO-12-$NH_2$ | <1.7 | 589 | 0.39 |
| PMO-14-$NH_2$ | 1.93 | 523 | 0.34 |
| PMO-16-$NH_2$ | 2.30 | 749 | 0.62 |
| PMO-18-$NH_2$ | 1,90 | 407 | 0.38 |
| PMO-12-R1 | <1.7 | 262 | 0.21 |
| PMO-14-R1 | <1.7 | 235 | 0.25 |
| PMO-16-R1 | 1.93 | 521 | 0.45 |
| PMO-18-R1 | 2.19 | 379 | 0.44 |
| PMO-12-R2 | <1.7 | 331 | 0.22 |
| PMO-14-R2 | <1.7 | 275 | 0.26 |
| PMO-16-R2 | 1.93 | 596 | 0.55 |
| PMO-18-R2 | 1.92 | 446 | 0.48 |

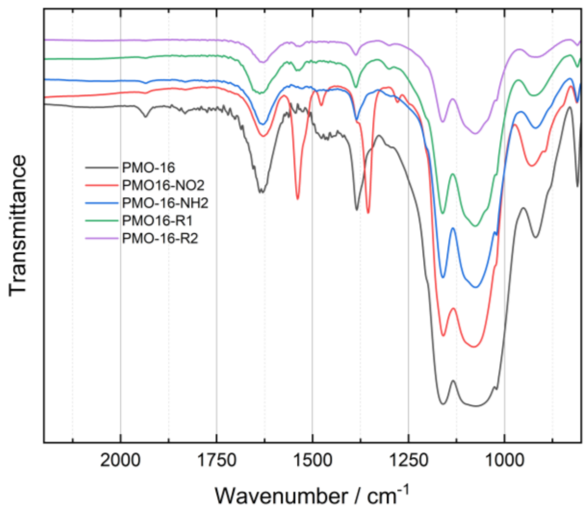


**Figure S5.** FTIR spectra of the functionalized PMO-16 series. (Spectra are vertically shifted for better visibility.).

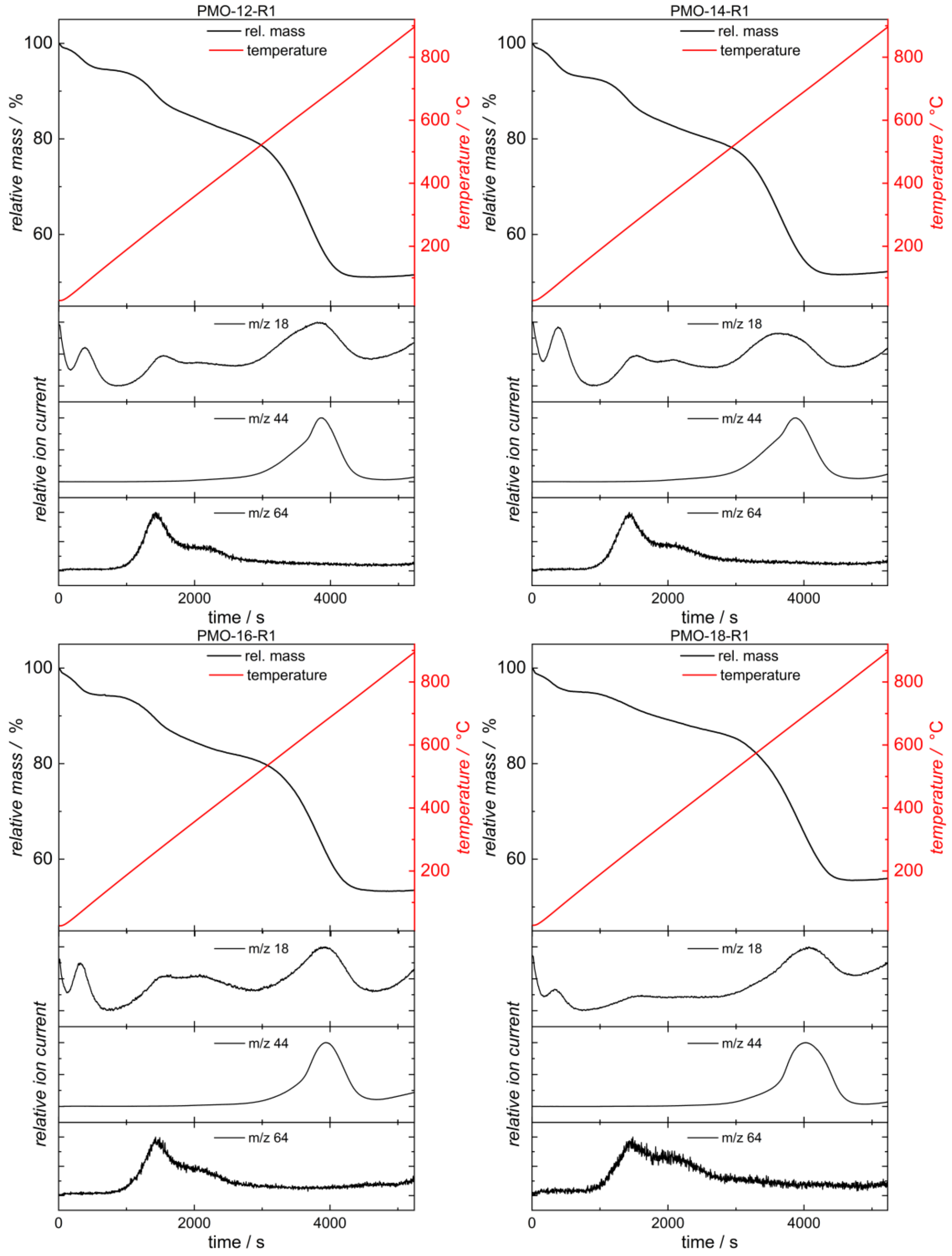


**Figure S6.** TGA-MS plots of functionalized PMO (R1).

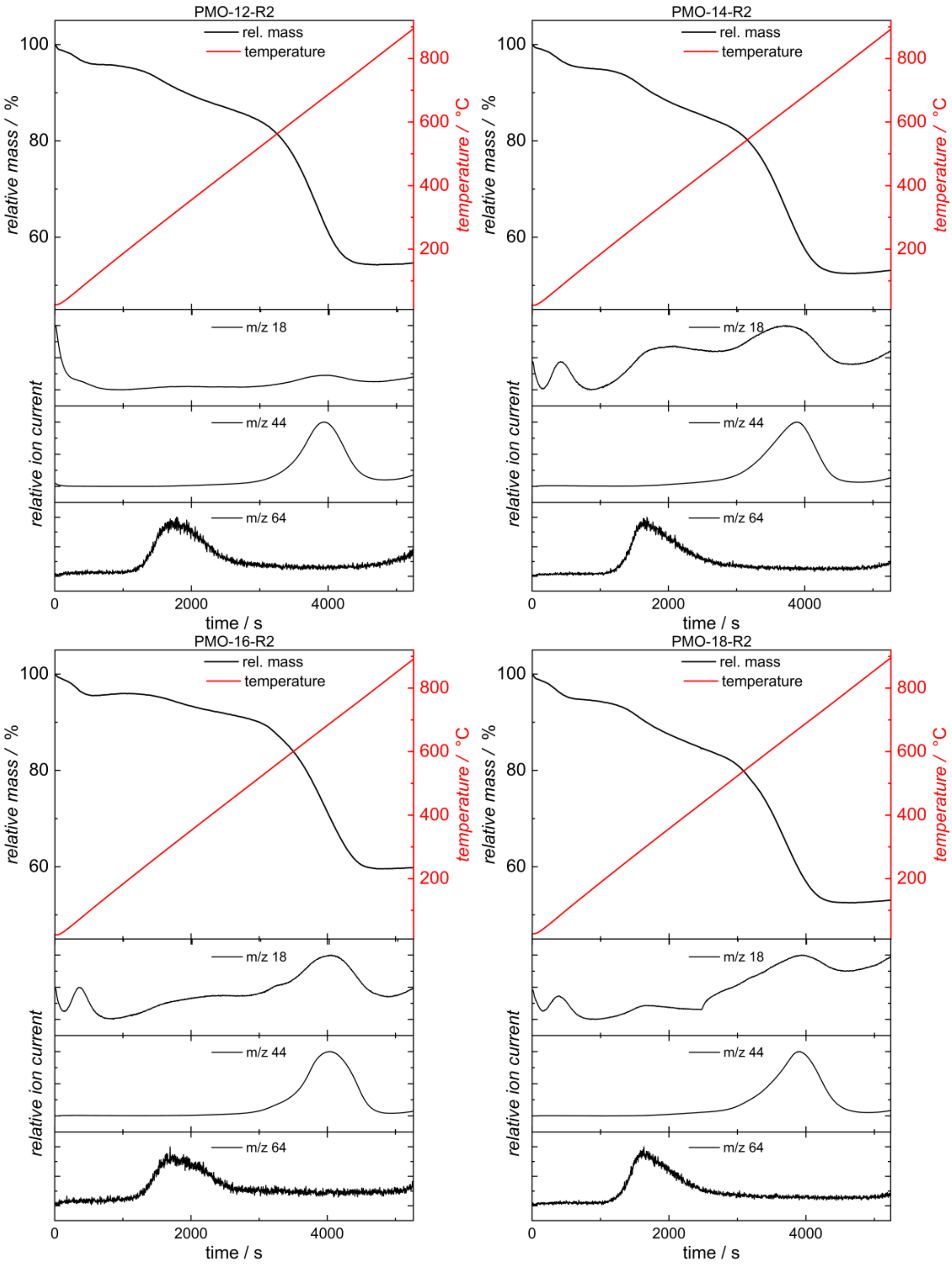


**Figure S7.** TGA-MS plots of functionalized PMO (R2).

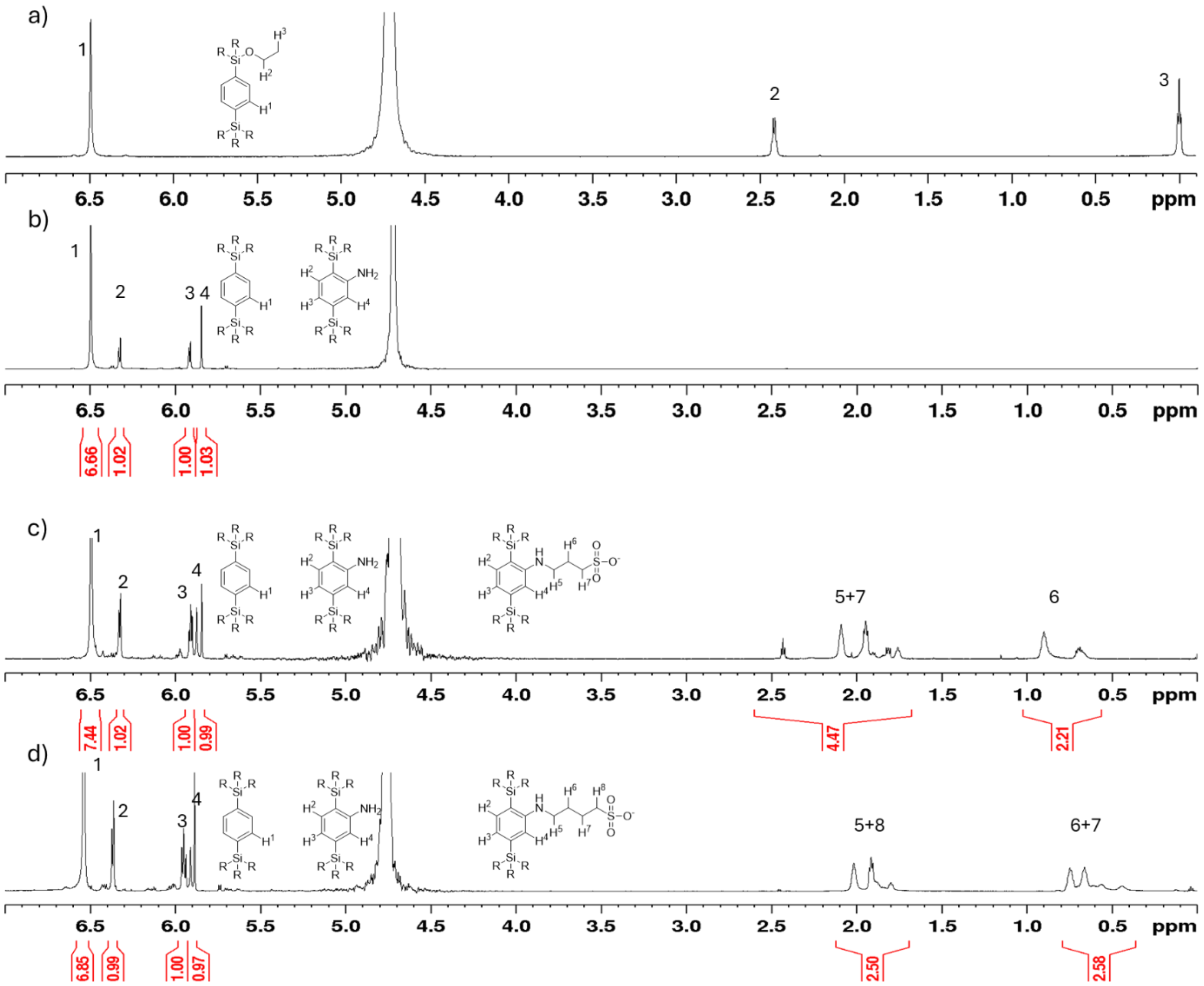


**Figure S8.** NMR spectra of digested (a) PMO-16, (b) PMO-16-$NH_2$, (c) PMO-16-R1, and (d) PMO-16-R2.

**Calculation of the degree of functionalization.** Integrals of the 1H NMR peaks were calibrated to the $H^3$ peak. The degree of functionalization was calculated from the fraction of the unfunctionalized ($H^1$) to the amino-functionalized ($H^3$) protons (**S1**). For the sulfonate function, the fraction of the aliphatic protons (for R1: $H^5$+ $H^6$+$H^7$; for R2: $H^5$+ $H^6$+$H^7$+ $H^8$) compared to the amino-functionalized protons ($H^3$) was used (**S2** and **S3**). The overall degree of functionalization is the product of these two (**S4**).

$$f\%(\mathrm{NH_2}) = 1 - \frac{\int \mathrm{H}^1/4}{\int \mathrm{H}^1/4 + \int \mathrm{H}^3} \quad \text{(S1)}$$

$$f\%(R1) = \frac{\int \mathrm{H}^{5+6+7}/6}{\int \mathrm{H}^3} \quad \text{(S2)}$$

$$f\%(R2) = \frac{\int \mathrm{H}^{5+6+7+8}/8}{\int \mathrm{H}^3} \quad \text{(S3)}$$

$$f\%(overall) = f\%(\mathrm{NH_2}) \cdot f\%(R) \quad \text{(S4)}$$

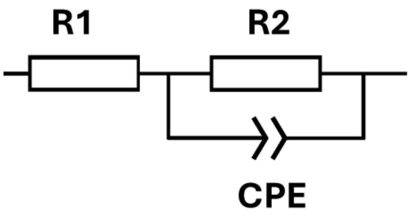


**Figure S9.** Equivalent circuit used for impedance data fitting.

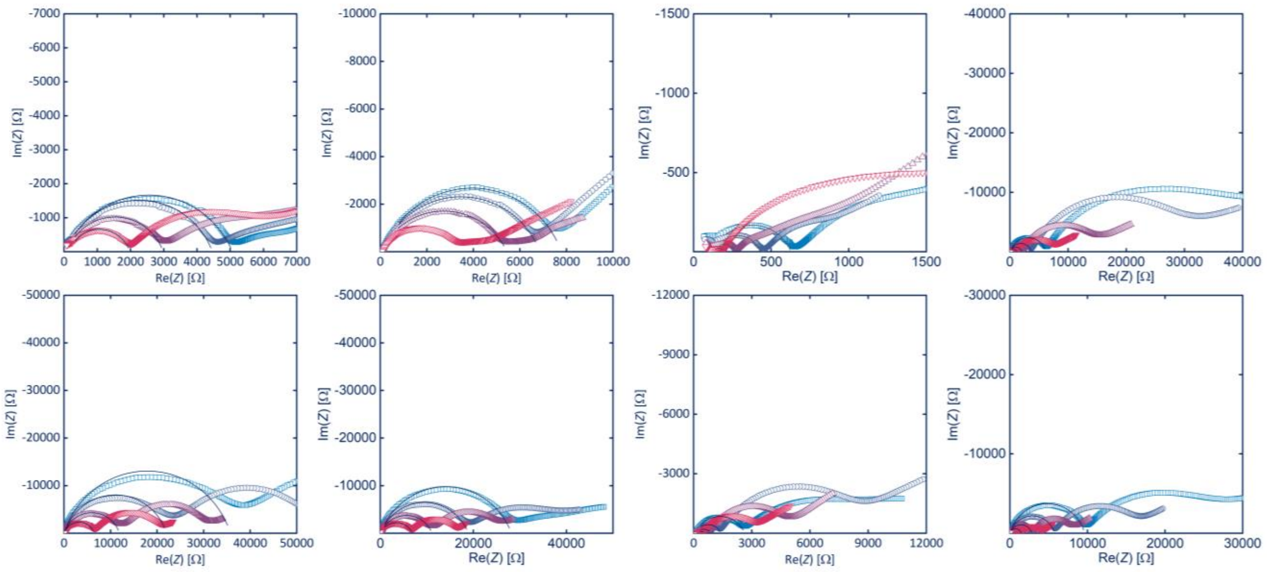

**Figure S10.** Nyquist plots for different pore sizes (left to right: PMO-12 to PMO-18) and different functionalization degrees (top: -R1; bottom: -R2).

**Table S2.** Proton conductivity and IEC of comparable silica-based materials and Nafion.

| material | T [°C] | rh [%] | σ [mS/cm] | IEC [mmol/g] | reference |
|---|---|---|---|---|---|
| sulfonated mesoporous silica MCM-41-MPTMS | 140 | 100 | 1.5 | 1.16 | [21] |
| functionalized mesoporous silica films | 25 | 20 | 5.4 | 2.3 | [23] |
| sulfonic acid-functionalized mesoporous silica | 25 | 100 | 7.2 | 1.1 | [24] |
| porous silica glass | 50 | 80 | 2.0 | - | [42] |
| Nafion 117 | 25 | 100 | 78 | - | [43] |
| Nafion 117 | 30 | 70 | 15 | 0.93 | [44] |

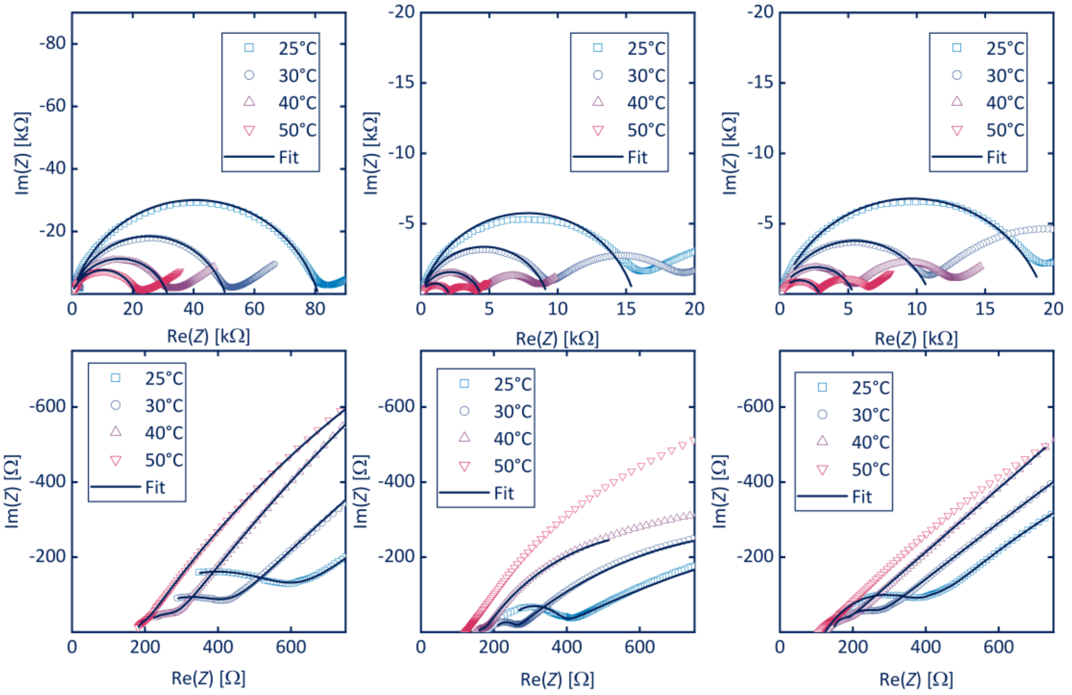


**Figure S11.** Nyquist plots for different functionalization degrees from 14% (top left) to 46% (bottom right).

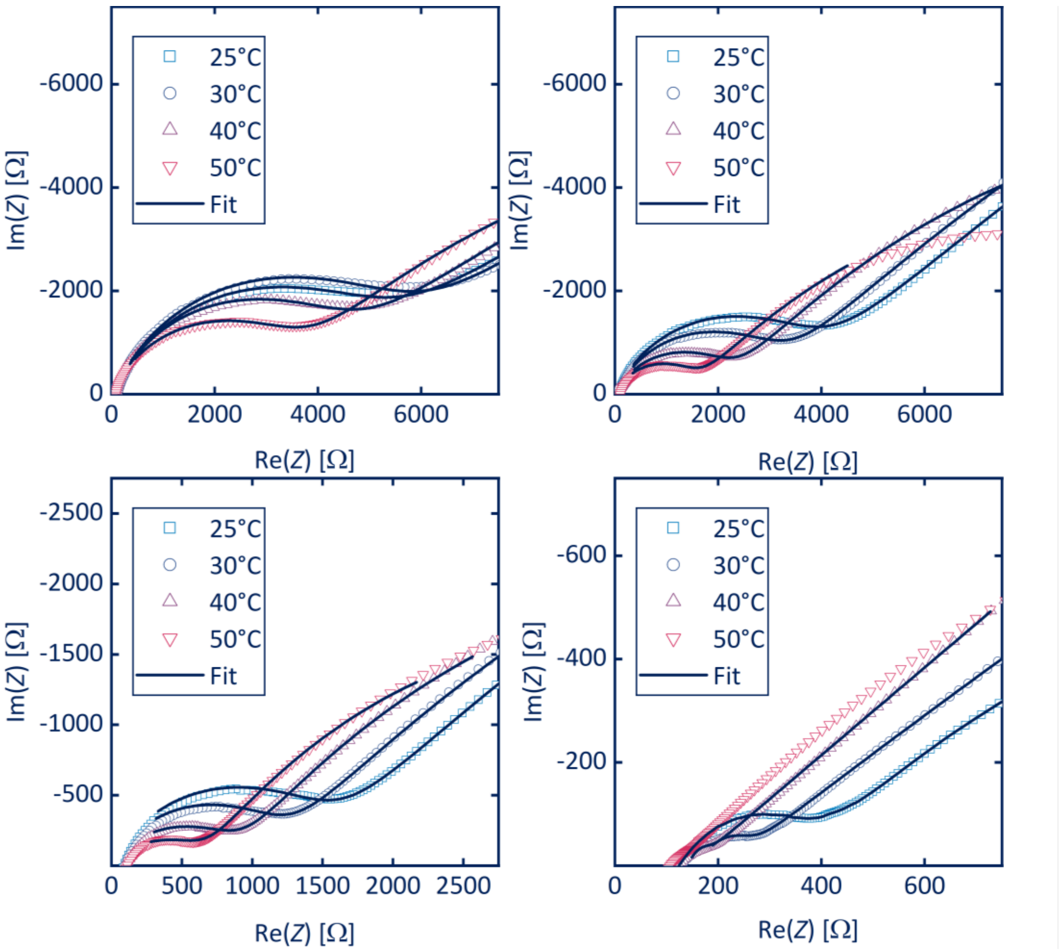


**Figure S12.** Nyquist plots for different humidities (top left—60 % r.h.; top right—70 % r.h.; bottom left—80 % r.h.; bottom right—90 % r.h.) at varying temperature.

## References


21. Marschall, R.; Bannat, I.; Caro, J.; Wark, M. Proton conductivity of sulfonic acid functionalised mesoporous materials. *Microporous Mesoporous Mater.* **2007**, *99*, 190–196, doi: 10.1016/j.micromeso.2006.08.037

23. Fujita, S.; Koiwai, A.; Kawasumi, M.; Inagaki, S. Enhancement of proton transport by high densification of sulfonic acid groups in highly ordered mesoporous silica. Chem. Mater. 2013, 25, 1584–1591, doi: 10.1021/cm303950u

24. Daiko, Y.; Kasuga, T.; Nogami, M. Pore size effect on proton transfer in sol-gel porous silica glasses. Microporous and Mesoporous Materials 2004, 69, 149–155, doi:10.1016/j.micromeso.2004.02.005.

42. Mikhailenko, S.; Desplantier-Giscard, D.; Danumah, C.; Kaliaguine, S. Solid electrolyte properties of sulfonic acid functionalized mesostructured porous silica. *Microporous and Mesoporous Materials* **2002**, *52*, 29–37, doi:10.1016/S1387-1811(02)00275-5.

43. Sone, Y.; Ekdunge, P.; Simonsson, D. Proton Conductivity of Nafion 117 as Measured by a Four-Electrode AC Impedance Method. *J. Electrochem. Soc.* **1996**, *143*, 1254–1259, doi:10.1149/1.1836625.

44. Sigwadi, R.; Dhlamini, M.S.; Mokrani, T.; Ṋemavhola, F.; Nonjola, P.F.; Msomi, P.F. The proton conductivity and mechanical properties of Nafion®/ ZrP nanocomposite membrane. *Heliyon* **2019**, *5*, e02240, doi:10.1016/j.heliyon.2019.e02240.
